# Zika Virus Infection During Research Vaccine Development: Investigation of the Laboratory-Acquired Infection *via* Nanopore Whole-Genome Sequencing

**DOI:** 10.3389/fcimb.2022.819829

**Published:** 2022-03-07

**Authors:** Eunsik Bang, Sujin Oh, Ho Eun Chang, Il Seob Shin, Kyoung Un Park, Eu Suk Kim

**Affiliations:** ^1^ Department of Medicine, Seoul National University College of Medicine, Seoul, South Korea; ^2^ Department of Laboratory Medicine, Seoul National University College of Medicine, Seoul, South Korea; ^3^ PHiCS Institute, Seoul, South Korea; ^4^ Department of Laboratory Medicine, Seoul National University Bundang Hospital, Seongnam, South Korea; ^5^ Department of Internal Medicine, Seoul National University College of Medicine, Seoul, South Korea; ^6^ Department of Internal Medicine, Seoul National University Bundang Hospital, Seongnam, South Korea

**Keywords:** whole genome sequencing, WGS, viral strain typing, laboratory-acquired infection, LAI

## Abstract

Zika virus (ZIKV) emerged as a serious public health problem since the first major outbreak in 2007. Current ZIKV diagnostic methods can successfully identify known ZIKV but are impossible to track the origin of viruses and pathogens other than known ZIKV strains. We planned to determine the ability of Whole Genome Sequencing (WGS) in clinical epidemiology by evaluating whether it can successfully detect the origin of ZIKV in a suspected case of laboratory-acquired infection (LAI). ZIKV found in the patient sample was sequenced with nanopore sequencing technology, followed by the production of the phylogenetic tree, based on the alignment of 38 known ZIKV strains with the consensus sequence. The closest viral strain with the consensus sequence was the strain used in the laboratory, with a percent identity of 99.27%. We think WGS showed its time-effectiveness and ability to detect the difference between strains to the level of a single base. Additionally, to determine the global number of LAIs, a literature review of articles published in the last 10 years was performed, and 53 reports of 338 LAIs were found. The lack of a universal reporting system was worrisome, as in the majority of cases (81.1%), the exposure route was unknown.

## Introduction

Zika virus (ZIKV) is a small, enveloped positive-strand RNA virus belonging to the *Flavivirus* genus of the Flaviviridae family (Lin et al., 2018). ZIKV was first isolated from non-human primates in 1947 and mosquitoes in 1948 in Africa (Musso and Gubler, 2016). After its discovery, reports of human ZIKV infections were less than 20, mostly from Yellow Fever serosurveys (Musso and Gubler, 2016). However, ZIKV infection emerged as a serious public health problem in 2007, when the first ZIKV outbreak occurred in the Federated States of Micronesia (Musso and Gubler, 2016). Since then, major ZIKV outbreaks have occurred in numerous Pacific islands, North and South America, and West Africa (Musso and Gubler, 2016). As of July 2019, 87 countries and territories have recorded autochthonous mosquito-borne transmission, distributed among Africa, Americas, South-East Asia, and Western Pacific Regions (Pielnaa et al., 2020). Although most ZIKV infections are asymptomatic or mild, some patients develop neurological abnormalities such as Guillain-Barre syndrome. Neonatal microcephaly, arthrogryposis, and ophthalmic changes can also be seen in infants with Congenital Zika syndrome (Freitas et al., 2020). For example, the incidence of neonatal microcephaly increased 20-fold in Brazil seven years ago due to a Zika outbreak (Lin et al., 2018).

Because of these concerns, the WHO declared the ZIKV a “Public Health Emergency of International Concerns”, and researchers have made numerous attempts to develop a vaccine. The ZIKV RNA genome encodes three structural genes (core C, membrane precursor prM, and envelope E), seven non-structural genes (NS1, NS2A, NS2B, NS3, NS4A, NS4B, and NS5), and an untranslated region (Lin et al., 2018). These genes, or proteins translated therefrom, are the targets of the various ZIKV vaccine candidates. Researchers attempted to modify viral capsid protein and ZIKV genomes to develop a live attenuated ZIKV vaccine, and some of the results showed increased neutralizing antibody titers (Shan et al., 2018; Xie et al., 2018). A purified inactivated vaccine derived from the Puerto Rican strain (PRVABC59) prevented ZIKV against ZIKV infection in mice and non-human primates (Abbink et al., 2016). Subunit vaccines such as mRNA-based and plasma-based DNA vaccines provide protection by expressing two virion surface glycoproteins: prM and E (Shan et al., 2018). As of December 2019, 18 known vaccine candidates are in various stages of preclinical and clinical development (Pielnaa et al., 2020). However, despite the success of these trials no vaccine for human use is yet available. Additionally, no ZIKV antiviral drugs have been found to treat ZIKV infection effectively for the moment (Poland et al., 2019; Pielnaa et al., 2020).

Furthermore, there is increasing concern regarding the possibility of laboratory-acquired ZIKV infections. To our knowledge, two laboratory-acquired ZIKV infections have been reported in the last ten years. In the first, which occurred in 2016, a researcher at the University of Pittsburgh stuck herself with a ZIKV-infected needle (Newsroom, 2016). The second happened in June 2017, where a mouse bite led to a laboratory-acquired ZIKV infection in a laboratory in Brazil (Talon de Menezes et al., 2020).

Current ZIKV diagnosis is performed by detecting its genome or through serological assays, which measure antibody concentration against viral proteins (Herrada et al., 2018). The former method is Real-time reverse transcriptase PCR (rRT-PCR), which is currently recommended to detect and quantify ZIKV viral RNA. The latter methods are Zika IgM Antibody Capture Enzyme-linked Immunosorbent Assay (Zika MAC-ELISA) and the plaque reduction neutralization test (PRNT), which detect IgM against ZIKV and the decrease of plaque-forming units in cell cultures, respectively.

For persons suspected of ZIKV infection, a positive rRT-PCR result can confirm the infection. However, due to reporting inaccuracies and a decrease in the level of viremia, a negative rRT-PCR result does not exclude ZIKV infection (Rabe et al., 2016). For serum specimens collected less than 7 days after the onset of symptoms, negative results for both the rRT-PCR and Zika MAC-ELISA are needed in order to rule out ZIKV infection (Rabe et al., 2016). PRNTs are performed when the Zika MAC-ELISA yields positive, equivocal, or inconclusive results. A negative PRNT result for a specimen collected more than 7 days after the onset of symptoms rules out ZIKV infection. However, for specimens collected less than 7 days after the onset of symptoms, negative results for both the PRNT and rRT-PCR are needed to exclude the possibility of infection (Rabe et al., 2016).

To summarize, there are two main disadvantages of serological testing that decrease test accuracy. First, there is the possibility of cross-reaction between IgM Ab from other flaviviruses, thus of non-specific reactions. Second, it is impossible to determine when the antibody response is insufficient (Herrada et al., 2018; Talon de Menezes et al., 2020). PCR-based methodologies are biased and dependent on the sequence of the primers. Thus, they are not useful for analyzing unknown pathogens. Additionally, both modalities have weaknesses in tracking pathogen evolution and the transmission route (Lin et al., 2018).

To overcome these limitations, whole-genome sequencing (WGS) using next-generation sequencing (NGS) technology has been suggested as a new tool in viral epidemiology research, especially for analyzing pathogen outbreaks. These methods can also be used on cultural isolates of bacteria and fungi, and can facilitate pathogen identification, the assessment of susceptibility to antimicrobials, and outbreak investigation, and surveillance (Cameron et al., 2020).

The potential value of genome sequencing for establishing the origin and tracking the evolution of viruses has been acknowledged by various studies in literature, and several examples of the application in clinical epidemiology have been reported (Wohl et al., 2016; Artika et al., 2020). One of the earlier examples was the phylogenetic analysis of human influenza A H5N1 in the 1997 Hong Kong outbreak (Wohl et al., 2016). Analysis of genome samples to understand the origins and evolution of the Makona variant that caused the 2014 West African Ebola virus epidemic is another example (Rasmussen and Katze, 2016). Recently, third-generation sequencing, also known as long-read sequencing, has been used in real-time analysis and surveillance of pathogen outbreaks (Kono and Arakawa, 2019). With its advantage of real-time nature and rapidness, nanopore sequencing enabled genotyping of a hospital outbreak of *Salmonella* sp. less than half a day and the development of genomic surveillance system for the Ebola outbreak in West Africa (Kono and Arakawa, 2019).

Here, we describe the case of a suspected ZIKV LAI in a male researcher who was working in a ZIKV research vaccine development laboratory. With this case, we tried to determine whether WGS is a powerful tool for conducting viral strain typing and determining the degree of relatedness. This project was approved by Seoul National University Bundang Hospital Institutional Review Board (SNUBH IRB), with IRB No. of B-2105-682-701. The waiver of informed consent was also approved by the SNUBH IRB.

## Materials and Methods

### Case Information

The laboratory worker completed laboratory safety training prior to the experiment. During the day of the experiment, he injected a highly concentrated live ZIKV strain into a mouse, followed by blood withdrawal and sacrifice of the mouse. During the procedure, he wore a surgical mask and gloves, as well as a lab coat. He did not wear protective goggles but reported no blood splashing during the procedure. A hand sanitizing protocol was strictly followed using soap. He reported that he engaged in unprotected sexual intercourse with his girlfriend between the day of the experiment and the day when symptoms first appeared. A week after the experiment (Day 7), he experienced chills and joint pain. On Day 9, he reported rashes on his extremities and trunk with pruritus; these symptoms worsened until Day 11. All symptoms had improved by Day 13. As ZIKV infection was suspected, he visited Seoul National University Bundang hospital, where real-time PCR testing was conducted. WGS was also conducted to verify the relationship between the patient and the ZIKV strains used for vaccine development.

### Specimens Used in This Study

Direct plasma, semen, and urine specimens were gathered from the patient and tested for ZIKV on Day 13, 28, 35, and 41 *via* real-time RT-PCR. Plasma and urine specimens from the patient’s partner were also gathered and tested to exclude the possibility of sexual transmission. The WGS procedure was performed when a specimen was positive according to real-time RT-PCR.

### Real-Time Reverse Transcriptase PCR Analysis of Patient Specimens

The first step in the analysis of each specimen was nucleic acid extraction, which was done using EasyMAG (Biomeriux, Marcy-l’Étoile, France). First, 500 μL of each specimen was mixed with 2.5 μL of the Internal Control, and then eluted to 25 μL. Then, amplification was conducted using a 7500 real-time PCR system (Thermo Fisher Scientific, Waltham, MA, USA). The PCR mix consisted of 5 μL of Master Mix A, 15 μL of Master Mix B, and 10 μL of template RNA or control. Distilled water was used as a negative control, and ZIKV-specific RNA was used as a positive control. The PCR conditions were as follows: 55°C for 20 minutes and 95°C for 2 minutes, followed by a program of 95°C for 15 seconds, 55°C for 45 seconds, and 72°C for 15 seconds for 45 cycles. To detect amplification of the ZIKV genome, FAM fluorescent dye was used with the threshold set to 0.1. To detect the amplification of internal control genes, JOE fluorescent dye was used with the threshold set to 0.05.

### Whole-Genome Sequencing Using Nanopore Sequencing Technology

The Multiplex PCR Protocol with a bioinformatics workflow, in this case using the MinION sequencer (Oxford Nanopore Technologies, Oxford, UK), was used for WGS of the patient sample. We used the protocol based on Quick et al.’s literature, “Multiplex PCR method for MinION and Illumina sequencing of Zika and other virus genomes directly from clinical samples” (Quick et al., 2017). The entire whole-genome sequencing process was performed at PHiCS Institute, Seoul, South Korea.

#### Primer Design and RNA Extraction

To produce ZIKV specific-binding primers, the ZIKV reference genome (NCBI reference sequence: NC_012532.1) was identified. Thirty-eight pairs of primer schemes were generated using Primal Scheme (primal.zibraproject.org, **Supplementary Table 1**). RNA was extracted from 200 μl of the urine specimen using the QIAamp Viral RNA Mini Kit (QIAGEN, Venlo, Netherlands), as per the manufacturer’s guidelines.

#### cDNA Synthesis

cDNA from the extracted RNA was synthesized using the High-Capacity RNA-to-cDNA™ Kit (Life Technologies Corporation, Carlsbad, CA, USA). In a 20 μL reaction, we used 1 μL of 20× RT Enzyme Mix, 10 μL of 2× RT Buffer Mix, 7 μl of the RNA sample, and 2 μl of DW. cDNA synthesis was performed with the following conditions: 37°C for 60 minutes followed by 97°C for 5 minutes and 4°C for 5 minutes. The solution was held at 4°C afterwards.

#### Multiplex PCR

For the multiplex PCR using the designed primers, we used AmpliTaq Gold 360 Master Mix (Applied Biosystems, Foster City, CA, USA). In a 25-μL PCR reaction, we used 12.5 μL of 2× Master Mix, 0.5 μL of the primers (10 pmol/μL), both forward and reverse, 2.5 μL of cDNA, and 9 μL of DW. The multiplex PCR was conducted under the following conditions: 95°C for 5 minutes, followed by a program of 95°C for 15 seconds, 60°C for 30 seconds, and 72°C for 60 seconds for 40 cycles, ending with 72°C for 7 minutes.

#### Cleanup and Quantification of Amplicons

For the quantification of amplicons, the contents were mixed with SPRI beads (Beckman Coulter, Brea, CA, USA) according to the manufacturer’s instructions, and then cleaned using nuclease-free water. Then, 1 μL of the cleaned product was quantified using the Quantus fluorometer (Promega, Madison, WI, USA). We quantified 38 obtained PCR products, which ranged from 5~30 ng/μL.

#### Library Preparation and Sequencing

A library was designed using the Amplicon by Ligation kit (Oxford Nanopore Technologies). Amplicons were pooled with a final amount of 200 fmol, and then subjected to DNA end-repair and cleanup processes. A sequencing adapter was ligated to the end-repaired amplicons, and 50 fmol of the prepared library was loaded onto flow cells. Sequencing was conducted using MinION (Oxford Nanopore Technologies).

#### Data Analysis

Data (FASTQ) derived from MinION were analyzed using the CLC Genomics Workbench 20.1 (Qiagen, Venlo, Netherlands). With the imported data, *de novo* assembly was first conducted. Contigs made from the *de novo* assembly were then read to the ZIKV reference genome (NCBI reference sequence: NC_012532.1). This process enabled extraction of a consensus sequence from the patient sample. The derived consensus sequence was then aligned with three different ZIKV strains. One of these was a ZIKV reference genome (NCBI reference sequence: NC_012532.1). The other two strains were KU955591.1 (ZIKV isolate ZIKV/A.africanus-tc/SEN/1984/41525-DAK) and KX446950.2 (ZIKV strain ZIKV/Aedes.sp/MEX/MEX_2-81/2016, complete genome), which were the ZIKV strains used in the vaccine development. A phylogenetic tree was also produced to clarify the relationship between known ZIKV strains and the patient sample. The ZIKV strains used for the phylogenetic tree are listed in **Table 1**. Maximum likelihood method was used to create the phylogenetic tree, and bootstrapping was conducted with the number of 100 replicates.

**Table 1:** ZIKV strain list used to produce the phylogenetic tree.

GenBank accession number	Strain Name	Percent identity (*via* BLAST)	Group
KU501217	8375	89.09%	A
KU870645	FB-GWUH-2016	89.22%	A
KX446950	ZIKV/Aedes.sp/MEX/MEX_2-81/2016	89.09%	A
KU647676	MRS_OPY_Martinique_PaRi_2015	89.18%	A
KU922923	MEX/InDRE/Lm/2016	89.18%	A
KU820897	FLR	89.18%	A
KX087102	ZIKV/Homo sapiens/COL/FLR/2015	89.18%	A
KU853013	Dominican Republic/2016/PD2	89.18%	A
KU926310	Rio-S1	88.86%	A
KU707826	SSABR1	89.41%	A
KU312312	Z1106033	89.28%	A
KX087101	ZIKV/Homo sapiens/PRI/PRVABC59/2015	89.37%	A
KU509998	Haiti/1225/2014	89.18%	A
KX051563	Haiti/1/2016	89.23%	A
EU545988	FSM	89.28%	A
LC002520	MR766-NIID	92.93%	B
NC_012532	MR 766	92.70%	B
KF383115	ArB1362	93.20%	B
KF268949	ARB15076	93.07%	B
KF268948	ARB13565	92.84%	B
KF268950	ARB7701	92.84%	B
KU963574	ZIKV/Homo sapiens/NGA/IbH-30656_SM21V1-V3/1968	97.04%	C
KU955591	Zika virus/A.africanus-tc/SEN/1984/41525-DAK	99.27%	D
KU955592	Zika virus/A.taylori-tc/SEN/1984/41662-DAK	99.27%	D
KU955595	Zika virus/A.taylori-tc/SEN/1984/41671-DAK	99.17%	D
KU681082	Zika virus/H.sapiens-tc/PHL/2012/CPC-0740	89.23%	A
KU955593	Zika virus/H.sapiens-tc/KHM/2010/FSS13025	89.28%	A
KU681081	Zika virus/H.sapiens-tc/THA/2014/SV0127- 14	89.00%	A
KJ776791	H/PF/2013	89.28%	A
KU866423	Zika virus/SZ01/2016/China	89.00%	A
KF383119	ArD158084	93.11%	B
KF383118	ArD157995	92.93%	B
KF383117	ArD128000	97.75%	C
KF383116	ArD7117	98.53%	C
HQ234501	ArD41519	99.27%	D
KX377336	P6-740	90.23%	B
KX694532	PLCal ZV	89.14%	A
KU501216	103344	89.14%	A

Details are given in the following order: GenBank accession No., strain/isolate name, and percent identity between the specimens. The strains were classified according to percent identity calculated *via* BLAST, describing the similarity between the patient specimen: Group A (percent identity < 90%), Group B (percent identity: 90~95%), Group C (percent identity: 95~99%), and Group D (percent identity: > 99%).

### Literature Analysis of Laboratory-Acquired Infections (LAIs) From 2011–2020

To determine the global number of LAIs, we performed a literature review of articles published in the period 2011–2020. We searched PubMed, ProMED-mail, and the American Biological Safety Association (ABSA) LAI database (https://my.absa.org/LAI) using the following keywords: ‘LAI’, ‘laboratory-acquired infection’, and ‘laboratory-acquired’. To ensure the accuracy of our analysis, i.e., to restrict it to LAIs that occurred recently in clinical or research laboratory settings, we excluded articles on LAIs in veterinary- or necropsy-based settings, as well as articles reporting cases before 2000. We also excluded articles that did not clearly describe the incident(s), those that retrospectively confirmed seroconversion, and surveys that were unable to match the incident(s) with the exposure route and causes. For each case, we recorded the cause of LAI, route of exposure, biological incident (act that led to LAI), type of laboratory, and biosafety level (BSL) of the laboratory.

## Results

### Real-Time Reverse Transcriptase PCR of Patient Samples

The test performed on Day 13 showed a negative plasma rRT-PCR result but a positive urine rRT-PCR result, with a cycle threshold (Ct) value of 24.73, confirming ZIKV infection. The test conducted on Day 28 showed negative results in the plasma, semen, and urine, as did those conducted on Day 35 and Day 41. The possibility of sexual transmission was excluded, as the patient’s girlfriend also had negative test results for all samples (urine and plasma).

### WGS Data Analysis

According to Epi2me, which is the data analysis platform used by Oxford Nanopore Technology, the average quality score of the reads was 11.7. This score is based on Oxford Nanopore Technology’s own algorithm, for which a score greater than 7 is considered to reflect high quality. In the *de novo* assembly, 140,879 contigs were produced with an average length of 441 bp. Of the 140,879 contigs, 130,758 were mapped in the ZIKV reference genome (NC_012532.1), with a coverage of 61.3%. The median coverage level of bases was 677.5, and the mean coverage level was 95383.59. Total of 10794 bases, 4195 bases were not covered. In the perspective of genes, the portions responsible for Glycoprotein E C-terminal domain, Glycoprotein E stem/anchor domain, NS2A, NS2B, Peptidase_S7 domain (part of NS3) were totally missing. The portions responsible for Protein C, Propeptide, Glycoprotein E central and dimerization domain, NS1 domain, DEAD-box domain (part of NS3), NS5 were partially sequenced. The portions responsible for membrane glycoprotein M, C-terminal helicase domain of NS3, NS4A, NS4B were fully sequenced.

Between NC_012532.1 and the patient sample, nine sequences were aligned, and the percent identity was 92.70%. Between KU955591.1 and the patient sample, nine sequences were aligned, which were almost identical to the former pairs. The calculated percent identity was 99.27%. Between KX446950.2 and the patient sample, 8 sequences were aligned, and the percent identity was 89.09%. From this data, we inferred that the patient sample was closer to the KU955591.1 strain, followed by NC_012532.1, and then KX446950.2.

**Figure 1:**
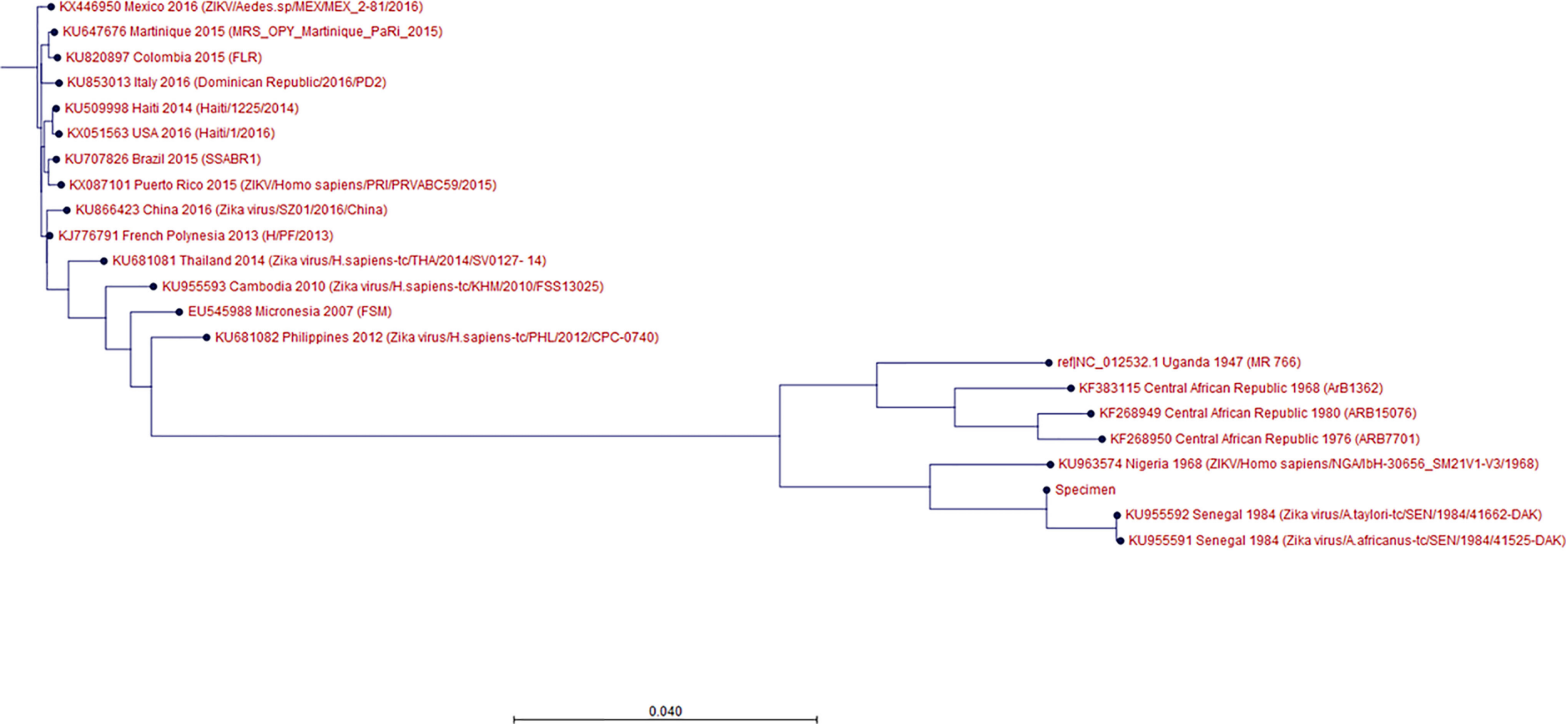
Phylogenetic tree showing the ZIKV strains and the specimen. Out of 31 ZIKV strains chosen, 17 are shown in this tree; the details are given in the following order: GenBank accession No., country, collection date, strain/isolate name. Maximum likelihood method was used to create the phylogenetic tree, and bootstrapping was conducted with the number of 100 replicates. ‘Specimen’ refers to the ZIKV derived from the patient’s specimen. KU955591, KX446950 are the strains from the patient’s vaccine lab. The specimen was most highly related to KU955591, followed by NC_012532.1 and KX446950.

The phylogenetic tree showed that the patient sample was closest to the KU955591, KU955592, and KU955595 ZIKV strains. This result was identical to that obtained by BLAST alignment, which showed the greatest proximity between KU955591 and the patient sample. The phylogenetic tree also showed that the distance between the patient sample and NC_012532.1 was closer than the distance between the patient sample and KX446950.2, consistent with the BLAST alignment data (**Figure 1**). To validate these results, we conducted BLAST alignment between the consensus sequence from the patient sample and the remaining ZIKV strains. The strains were classified into four groups based on the percent identity (< 90%, between 90~95%, between 95~99%, and above 99%), in an identical manner to the classification produced by the phylogenetic tree. The strains most closely related to the patient sample were KU955591 and KU955592 (both 99.27%). The other BLAST results are listed in **Table 1**.

### Literature Analysis of LAIs for the Period 2011–2020

**Figure 2:**
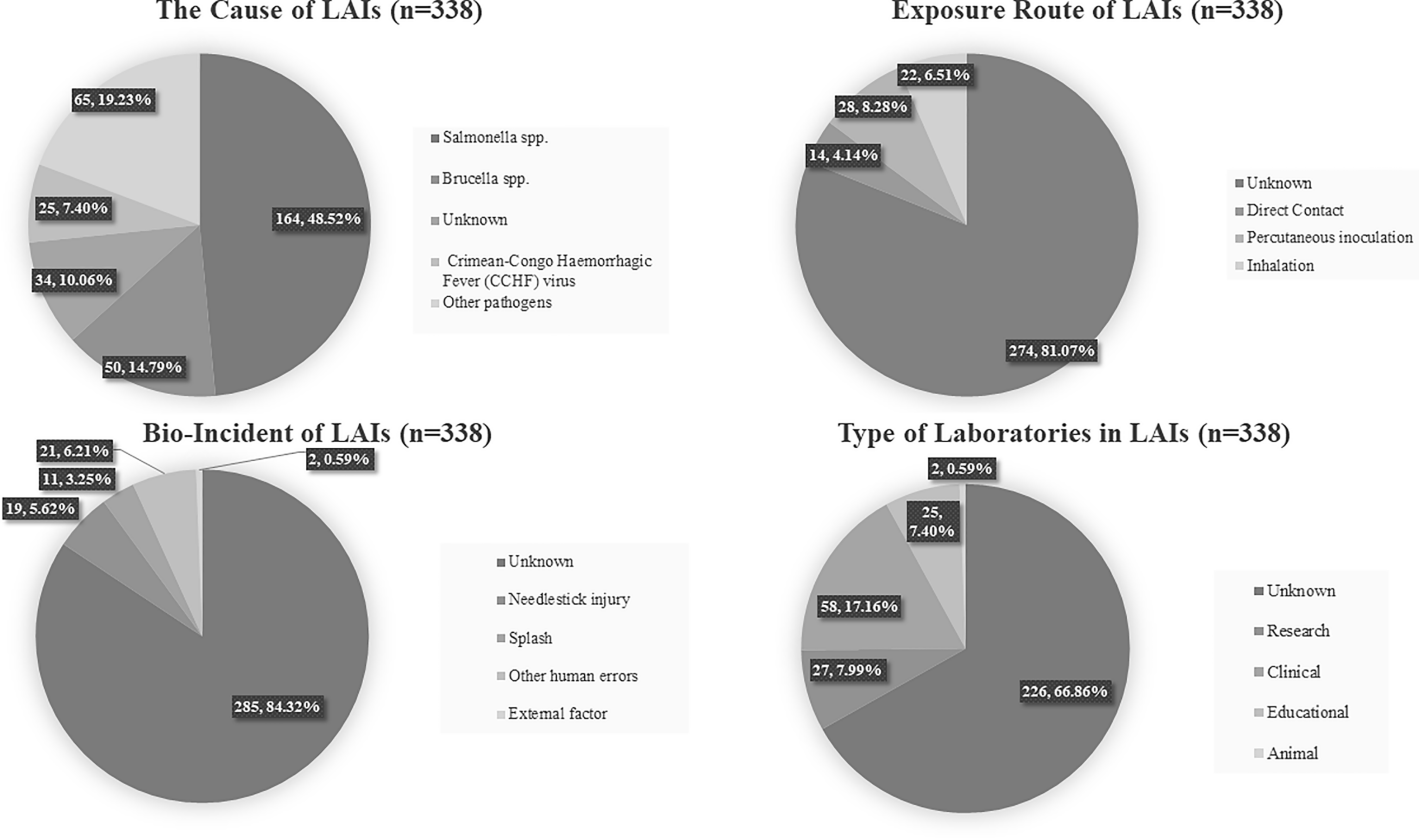
Simplified pie chart showing the results of our analysis of 338 LAIs from previous reports. The causative agent, exposure route, bio-incident, and type of laboratory are given.

Our brief review of LAIs that occurred from 2011–2020 uncovered 53 reports of 338 LAIs, including case reports and summary reports (Belchior et al., 2011; Britton et al., 2011; Buitrago et al., 2011; Centers for Disease Control and Prevention, 2011; Hitt, 2011; Horvat et al., 2011; Kaiser, 2011; Omer et al., 2011; Wunschel et al., 2011; Barrett 2012; Felinto de Brito et al., 2012; Lam et al., 2012; McCollum et al., 2012; Sayin-Kutlu et al., 2012; Seetharam et al., 2012; Centers for Disease C, Prevention, 2013; Cullen et al., 2013; Rodrigues et al., 2013; Traxler et al., 2013; Cullen et al., 2014; Hartady et al., 2014; Herriman 2014; Riyesh et al., 2014; Sheets et al., 2014; Barker et al., 2015; Benoit et al., 2015; Hsu et al., 2015; Joyce et al., 2015; Korioth-Schmitz et al., 2015; Langford et al., 2015; Vranes et al., 2015; Aebischer et al., 2016; Alexander et al., 2016; Campos-Macías et al., 2016; Duman et al., 2016; Garofolo et al., 2016; Leblebicioglu et al., 2016; Lee et al., 2016; Motlagh et al., 2016; Nedelman, 2016; Wurtz et al., 2016; Bienek et al., 2017; Knight 2017; Smith et al., 2017; Soria et al., 2017; Pomerleau-Normandin et al., 2018; Choucrallah et al., 2019; O’Neill, 2019; Qasmi and Khan, 2019; Sloan-Gardner, 2019; Whitehouse et al., 2019; Sharp et al., 2020; Talon de Menezes et al., 2020). Of these, 304 LAI cases were derived from 26 different pathogens, and 34 cases were from unknown pathogens. Among the known pathogens, *Salmonella* spp. was the most frequent cause of LAI; it was responsible for 164 LAIs, while 50 LAIs were due to *Brucella* spp. and 25 to Crimean-Congo hemorrhagic fever (CCHF) virus. The exposure routes included direct contact, percutaneous inoculation, and inhalation, although in the majority of cases (81.1%) the exposure route was unknown. The causative incidents that led to LAI included needlestick injury (6%), accidental splash (3%), and other human errors (6%), although unknown incidents comprised the majority of cases (84%). The incidents occurred in clinical (17%), research (8%), and educational (7%) laboratories, although in the majority of cases the laboratory type was unknown (67%) (**Figure 2**).

## Discussion

Before interpreting our rRT-PCR and WGS results, we reviewed the possible transmission routes of ZIKV, as well as the ZIKV infection history in Korea, to exclude the possibility that the patient was infected outside the ZIKV vaccine laboratory.

The most well-known mode of ZIKV transmission is *via* mosquitoes. In Africa, *Aedes africanus, Aedes lutocephalus, Aedes furcifer*, and *Aedes vittatus* are known as enzootic vectors, whereas nearly all known urban ZIKV outbreaks are linked to *Aedes aegypti* mosquitoes (Gregory et al., 2017). Recent cases showed that *Aedes albopictus* is a potent vector for ZIKV (Leung et al., 2015). ZIKV was discovered in 1947 and identified as an arthropod-borne virus. However, following an initial report that suggested the possibility of sexual transmission of ZIKV in 2008, cases of symptomatic male-to-female, male-to-male, female-to-male, and asymptomatic male-to-female transmission have been documented (Gregory et al., 2017). Furthermore, maternal ZIKV infection is associated with adverse fetal and infant outcomes, such as microcephaly, brain abnormalities, and visual/hearing deficits (Gregory et al., 2017).

Although the present study was limited in terms of our inability to identify the exposure route, and by the lack of information regarding the patient’s laboratory environment, a number of aspects indicate that this was a case of laboratory-acquired ZIKV. Our rationale is as follows. First, Korea is not an endemic region of ZIKV. In Korea, *Aedes albopictus* is now common due to warmer winter temperatures and rapid urbanization (Lee et al., 2020). However, there are still no indigenous cases of Zika, chikungunya, and dengue fever reported in Korea (Im et al., 2021). As of November 2020, 33 ZIKV cases were reported in Korea, all of which were imported. Second, the possibility of an imported ZIKV infection can be excluded, as neither the patient nor his girlfriend reported traveling to ZIKV endemic sites. Sexual transmission can also be excluded, as the rRT-PCR amplification pattern was not indicative of ZIKV, either in the patient’s semen or his girlfriend’s samples. Given these findings, combined with the status of the patient as a researcher working in ZIKV research vaccine development, laboratory infection is a reasonable cause of this case.

Even though rRT-PCR showed that the patient was infected with ZIKV, based on a positive urine result, this finding alone cannot explain the transmission of the Zika strain used for vaccine development to the patient. Thus, WGS was used to facilitate identification of the link between the ZIKV strain used in vaccine development and that present in the patient’s urine. The comparison between the consensus sequence and the other ZIKV strains was successful, as both the BLAST alignment and phylogenetic tree showed that KU955591.1 was the strain most closely related to that in the patient’s sample. From this result, it can be inferred that the ZIKV strain in the patient’s sample was most likely transmitted from the ZIKV strain used for vaccine development. The results of the phylogenetic tree were cross-confirmed by BLAST alignment with the ZIKV strains used in the tree. Although both KU955591.1 and KU955592 showed a percent identity of 99.27% with the patient’s consensus sequence, we considered KU955591.1 to be the source of infection, since this strain was used in the ZIKV research vaccine development lab in which he worked.

Given these results, we believe that this case demonstrates the strength of WGS for viral strain typing and determination of relatedness between strains. By directly comparing the consensus sequence from the patient sample to known ZIKV strains, we were able to quantify the similarity between sequences, and thus comprehensively assess the relatedness. As the rRT-PCR and other conventional modalities cannot show the transmission route of an infection, this case highlights the importance of WGS for virus strain typing. Since strain typing is crucial for controlling an outbreak of infection, WGS appears to be a valuable epidemiological tool.

The limitation of our WGS procedure lies in the coverage of the sample genome. Although we were able to produce a high-quality consensus sequence, only 61.3% of the total consensus sequence was revealed; the remainder is still unknown. These unknown portions could have two explanations. First, if the infection was transmitted in a laboratory, the viral load could have been lower than that required for vector transmission. We used PCR to amplify this low viral load, which could also have led to low-quality contigs. Second, the portions that had low contig counts or were of low quality could have been excluded by MinION during sequencing, such that they remained unknown.

If we repeated the sequencing with MinION, we might have filled in the missing portion and expanded the coverage. However, in the diagnostic perspective, we thought a rough sequencing with the single MinION process was adequate to determine the degree of relatedness and conduct strain typing. Although some genes in ORF were missing, we could fully sequence the portion responsible for membrane glycoprotein M, C-terminal helicase domain of NS3, NS4A, NS4B, with multiple other genes being partially sequenced. This result implies that we could compare multiple genes with a single sequencing, similar to Multilocus Sequence Typing (MLST). Also, with the consensus sequence derived from a single sequencing, we could earn the consistent result in BLAST alignment and phylogenetic tree analysis.

Compared with traditional Sanger sequencing, WGS shows the strength in time-effectiveness and higher accuracy. The ORF region of ZIKV is commonly used in strain typing, with our experiment and Haddow et al.’s experiment of evaluating relationship between ZIKV strains responsible for outbreaks being an example (Haddow et al., 2012). Since the size of ZIKV ORF region is much longer than the read length limit of Sanger sequencing, multiple targets should be designated to analyze this ORF region using Sanger sequencing, and this greatly decreases the accuracy and the time-effectiveness. Also, Sanger sequencing is known for low sensitivity, and has lower ability of detecting single nucleotide variant because of low read depth and coverage, leading to lower accuracy of alignment process using raw data (Behjati and Tarpey, 2013). In this manner, it would be unrealistic for Sanger sequencing to earn the same quality of raw data and consensus sequence compared with WGS, and massive time for repetition of sequencing would be required for Sanger sequencing to produce the same quality of data. In conclusion, we think WGS has advantage in quantifying the similarity between the sequences compared to Sanger sequencing, since WGS has lower limit of detection and more read depth.

Our results are promising due to the characteristics of MinION. Currently, MiSeq (Illumina, San Diego, CA, USA), Ion Torrent NGS (Thermo Fisher Scientific), MinION (Oxford Nanopore Technologies), and the PacBio Sequel System (Pacific Biosciences, Menlo Park, CA, USA) are the major DNA sequencing platforms. Illumina and Ion Torrent NGS were the earlier sequencing method and are predominantly being used in clinical setting (Vestergaard et al., 2021). Since these are short-read sequencers, their read lengths are shorter than those of the other sequencers. For example, MiSeq has a read length of 150 bp, whereas Ion Torrent NGS has a read length up to 600 bp (van Dijk et al., 2018). Whereas Illumina is based on cyclic reversible dye chain termination, Ion Torrent sequencing technology is based on the incorporation of a DNA template from a prepared library together with a single bead (Vestergaard et al., 2021).

MinION and PacBio Sequel System are the examples of long-read sequencing, which is known as the third-generation sequencing and has been developing for the past few years. Long-read sequencing techniques are characteristic in the generation of long reads (Vestergaard et al., 2021). For example, MinION and PacBio Sequel System have a read length of up to 2M and 100K bp, respectively (van Dijk et al., 2018).

Since long-read sequencers bypass the DNA amplification, they have the potential of avoiding inherited errors from the amplification and reducing the transition time from sample acquisition to sequencing (Vestergaard et al., 2021). Further, the pocket-sized MinION sequencers have increased portability (van Dijk et al., 2018). The speed of long-read sequencers could be useful when investigating acute viral infection, and this advantage is augmented by the flexibility that such portable devices provide. However, one drawback is that the MinION has a relatively high error rate. Despite this, we found sequencing with the MinION to be sufficiently accurate for classification of ZIKV strains, as well for determining the degree of relatedness between them. As the MinION and other long-read sequencing techniques are continually being developed to increase the read length and decrease the error rate, the application of WGS for investigating and monitoring outbreaks is promising. Further studies using other viral strains are needed to validate WGS as a useful tool in viral infection investigation.

This case also highlights the importance of LAI prevention and surveillance. The patient was infected with ZIKV even though they reported that they followed the recommended precautions such as wearing surgical gloves, a protective mask, and lab coat, as well as following hand sanitizations protocol. This shows that standard precautions do not preclude the possibility of LAI. The WGS data indicate that direct contact occurred between the patient and the ZIKV used for vaccine development. This case emphasizes the need for more caution when handling high-risk pathogens like ZIKV, as well for thorough instruction. This is especially important given that review articles of LAIs consistently describe a high rate of human error. One positive aspect of this case of laboratory-acquired ZIKV infection is the promptness with which it was reported, which helped to prevent secondary infection.

Some reviews of LAI research have reported that LAIs are decreasing due to an increase in laboratory safety precautions in laboratories worldwide (Rodrigues et al., 2013). However, our analysis of the literature indicated that LAIs are still a major concern for researchers and laboratory technicians, as more than 300 cases of LAI were reported in the last 10 years. Furthermore, since we excluded several LAI cases in animal shelter and necropsy settings, and the ones that didn’t have compatible causes, the actual number of LAIs is likely higher than what is reported here. Furthermore, if we expanded the criteria to ‘laboratory exposure’, rather than ‘confirmed LAI’, the number of cases would have been higher.

The most critical problem regarding LAIs is the lack of a universal reporting system. Laboratories are often reluctant to participate in research on LAIs, which can undermine the accuracy of LAI prevalence data. For instance, only 23 of the 119 (19%) laboratories contacted answered a survey conducted by Wurtz (Wurtz et al., 2016). This reluctance was also evident in our literature review, as data on exposure route, biological incident, and laboratory type were not available in over 60% of cases. A transparent LAI reporting system should reduce the likelihood of secondary infection resulting from LAIs, and likely also the incidence of LAIs in laboratories worldwide. We recommend that the causative agent, possible route of exposure, possible incident resulting in LAI, type of laboratory and BSL, and prevention procedures be promptly reported when an LAI occurs.

Prevention procedures are important in preventing LAIs, and are generally based on the mechanisms by which an LAI could occur. For example, for ZIKV, punctures and cuts from contaminated needles and other sharp objects, along with compromised skin that could contact contaminated materials, are the most likely routes of exposure to ZIKV (Traxler et al., 2013). Compliance with standard biosafety practices, such as storing potentially infectious samples in controlled places, working at the appropriate BSL for the task being performed, and following arthropod containment guidelines should be emphasized to prevent laboratory infection (Traxler et al., 2013).

To our knowledge, laboratory-acquired COVID-19 (SARS-CoV-2) infection has not been reported. As of November 2020, Choy et al. reported no cases of COVID-19 LAI, and there were no literatures of COVID-19 LAI since then (Choy, 2020). However, millions of diagnostic tests have been conducted in clinical labs to verify COVID-19 (SARS-CoV-2) infection, and attempts are being made by numerous research laboratories to develop and polish more efficient COVID-19 treatments. Situations like that described in the present report emphasize the importance of biosafety inspections when dealing with COVID-19, both in clinical and laboratory settings. SARS-CoV-2 predominantly spreads *via* the respiratory tract and through close contact. The main transmission route is droplet transmission, as the viruses from the infected respiratory tract can directly infect mucous membranes of people within 2m proximity (Wu et al., 2021). Some evidence of airborne and fecal-to-oral transmission, but clinical cases of these mechanisms have not been reported (Wu et al., 2021). Other routes such as sexual, vertical transmission, and breastfeeding are the possible concerns of COVID-19 transmission (Wu et al., 2021).

The main difference between the risk of contracting the ZIKV and COVID-19 in a laboratory setting lies in the transmission vector. Sharp objects contaminated with the ZIKV are the main transmission vector, whereas aerosols, as in clinical settings, are the most important transmission vector for COVID-19, even in a laboratory setting. Therefore, laboratory workers should avoid experimental procedures that produce aerosol or droplets (Agalar and Ozturk Engin, 2020). Furthermore, such workers should 1) follow procedures that constitute Good Microbiological Practices and Procedures (GMPP) during blood sampling, 2) perform procedures in an approved biosafety cabin or primary containment device, and 3) use disinfectants such as sodium hypochloride, ethanol, hydrogen peroxide, quaternary ammonium compounds, and phenolic compounds (Agalar and Ozturk Engin, 2020).

As our review indicates, LAIs can occur in every type of laboratory. However, the importance of biosafety measures is getting higher, especially in vaccine development. As the number of researchers working to develop novel vaccines for both the ZIKV and COVID-19 increases, the chances of laboratory infection escalate. We hope that this case will demonstrate that laboratory infection can occur without the patient’s awareness; the patient in the present study did not report being cut by a needle or being bitten by a mosquito. To prevent laboratory infections, appropriate laboratory safety measures should be followed during all laboratory procedures.

## Data Availability Statement

The data analyzed in this study can be accessed in Sequence Read Archive (SRA), with the accession no. of PRJNA798536. (https://www.ncbi.nlm.nih.gov/sra/?term=PRJNA798536).

## Ethics Statement

The studies involving human participants were reviewed and approved by Institutional Review Board of Seoul National University Bundang Hospital. Written informed consent for participation was not required for this study in accordance with the national legislation and the institutional requirements. Written informed consent was not obtained from the individual(s) for the publication of any potentially identifiable images or data included in this article.

## Author Contributions

Conceptualization, EB, KP, and EK. Methodology, EB, HC, IS, and KP. Investigation, EB. Data curation, EB, HC, and IS. Writing—original draft preparation, EB. Writing—review and editing, EB, SO, EK, and KP. Visualization, EB. Supervision, EK and KP. All authors have read and agreed to the published version of the manuscript. All authors contributed to the article and approved the submitted version.

## Conflict of Interest

The authors declare that the research was conducted in the absence of any commercial or financial relationships that could be construed as a potential conflict of interest.

## Publisher’s Note

All claims expressed in this article are solely those of the authors and do not necessarily represent those of their affiliated organizations, or those of the publisher, the editors and the reviewers. Any product that may be evaluated in this article, or claim that may be made by its manufacturer, is not guaranteed or endorsed by the publisher.
