# Supplementary material for: Zika Virus Infection During Research Vaccine Development: Investigation of the Laboratory-Acquired Infection via Nanopore Whole-Genome Sequencing
Source: Front Cell Infect Microbiol. 2022 Mar 7;12:819829. doi: 10.3389/fcimb.2022.819829 (PMC8936174; doi:10.3389/fcimb.2022.819829)
Supplement: Supplementary file 1 [file Table_1.docx]

**Supplementary Table 1.** 38 pairs of ZIKV specific binding primers, based on ZIKV reference genome (NC_012532.1). 38 pairs of primer schemes were created via Primal Scheme.

| Primer Name | Sequence (5' --> 3') | Length | Primer Name | Sequence (5' --> 3') | Length |
| --- | --- | --- | --- | --- | --- |
| Zika_1_F | CTGACTCAGACTGCGACAGTTC | 22 | Zika_1_R | GCATGGCAGCCAGATCTTTCTT | 22 |
| Zika_2_F | GCCATCACTGGGTCTCATCAAT | 22 | Zika_2_R | GCACCAACAATCGACGTCATCT | 22 |
| Zika_3_F | AGATCATGGATCTTGGACACATGTG | 25 | Zika_3_R | TTGCTGACTCCTATGCACCTGA | 22 |
| Zika_4_F | TTGGCTTTTGGGAAGCTCAACG | 22 | Zika_4_R | GCTCCCTTTGCCAAAAAGTCCA | 22 |
| Zika_5_F | GGTGAAGCCTACCTTGACAAGC | 22 | Zika_5_R | CCTTGTGAACCAACCAGTGCTT | 22 |
| Zika_6_F | GGTTTTGGAAGCCTAGGACTTGA | 23 | Zika_6_R | GCTGCGGTACACAAGGAGTATG | 22 |
| Zika_7_F | GATGGATGGTGCAAAGGGAAGG | 22 | Zika_7_R | TGGTGGGTGATCTTCTTCTCCC | 22 |
| Zika_8_F | ACCCCGTAATCACTGAAAGCAC | 22 | Zika_8_R | AGATCAACACTCCCCCTAAGGC | 22 |
| Zika_9_F | TGTCCTGGTTCTCACAAATTCTCA | 24 | Zika_9_R | CGTTGAGCTCCCCTTCTACTGA | 22 |
| Zika_10_F | GTAGATTGGCAGCAGCAGTCAA | 22 | Zika_10_R | AGTGTGAAATACCCCGAACCCA | 22 |
| Zika_11_F | AACAGCTTTGTCGTGGATGGTG | 22 | Zika_11_R | ATTTGGGTCCTGTAGCCCTCTC | 22 |
| Zika_12_F | CACACATTGTGGACAGATGGAATAGA | 26 | Zika_12_R | TCAGTTGATCCTGCAGTCACCA | 22 |
| Zika_13_F | CTGGGCTAAAGATGGCTGTTGG | 22 | Zika_13_R | TTTCACGGGGTGTCCAATTAGC | 22 |
| Zika_14_F | ACACTGGAGGAGATGTAGCTCA | 22 | Zika_14_R | TAGTCCCAGGGCCATGACAAAT | 22 |
| Zika_15_F | CACTGACAACATCACCTTGGCA | 22 | Zika_15_R | TGACAATTAGCAGACCAACCGC | 22 |
| Zika_16_F | CCCTAGCGAAGTACTCACAGCT | 22 | Zika_16_R | CCATAGCGCACCACTCCTTTTT | 22 |
| Zika_17_F | TCATACTCAAGGTGGTCCTGATGA | 24 | Zika_17_R | GTCTGGATGTTCCTCGCTCTCT | 22 |
| Zika_18_F | ACTGTGGTCCATGGAAGCTAGA | 22 | Zika_18_R | CCAGCTCCAGGATGCAAGTCTA | 22 |
| Zika_19_F | TTAGTGCCATCACCCAAGGGAG | 22 | Zika_19_R | TGTGAAGTGGGCCTCATCCATA | 22 |
| Zika_20_F | TCGACTTAATGTGCCATGCCAC | 22 | Zika_20_R | CTGTATGACCCGTTTTCCAGCC | 22 |
| Zika_21_F | TGGGTGACGGATCATTCTGGAA | 22 | Zika_21_R | TGCATGGTCTTCGTCAGTCTCT | 22 |
| Zika_22_F | AGAGAGTCATTCTGGCTGGACC | 22 | Zika_22_R | TTATGGTGTTGTTGGTCGTGCC | 22 |
| Zika_23_F | GAGATCTTCCTGTTTGGCTGGC | 22 | Zika_23_R | TCTCTAGGGTCTCCGGCAATTG | 22 |
| Zika_24_F | CCAGGAAGCCATTGACAACCTC | 22 | Zika_24_R | GCGGTAATCAAGCCCAGAAGAC | 22 |
| Zika_25_F | TCCTATTGCTGGTGGTGCTCAT | 22 | Zika_25_R | GCATCCCTTTGCCCATACCAAA | 22 |
| Zika_26_F | AACTTTCATTACCCCAGCCGTC | 22 | Zika_26_R | TCTCCACTTGGGGGTCAATTGT | 22 |
| Zika_27_F | ACATGTACTTGATCCCAGGGCT | 22 | Zika_27_R | CCTTCCATTTCTCTCCCAGGGT | 22 |
| Zika_28_F | AGGGGAAGTTACTTGGCTGGAG | 22 | Zika_28_R | TCTTGAACTTTGCGGATGGTGG | 22 |
| Zika_29_F | AGTGCAAAGCTGAGATGGTTGG | 22 | Zika_29_R | TCATAGTGCTGGTGTATGGGCA | 22 |
| Zika_30_F | CTGAAGTGGAAGAAGCACGGAC | 22 | Zika_30_R | TCACTGCGGATCCTTTCAATGC | 22 |
| Zika_31_F | GGAGGATGTGAATCTCGGCTCT | 22 | Zika_31_R | CACAACCAGGAAGAGACCATGC | 22 |
| Zika_32_F | TGGTCAGCAAAGAGTTTTCAAGGA | 24 | Zika_32_R | AGCCCCTAGCCACATATACCAG | 22 |
| Zika_33_F | GAGGAGAGTGCCAGAGTTGTGT | 22 | Zika_33_R | TCCCTTTTTCAGCTGGTCTAAGGA | 24 |
| Zika_34_F | ACCAACCAAATGGAGAAAGGGC | 22 | Zika_34_R | TCCACTCTTGTGTGTCCTTCCT | 22 |
| Zika_35_F | AGATGATTGCGTTGTGAAGCCA | 22 | Zika_35_R | ACCAGGTAGTTCTCCCAGTTGG | 22 |
| Zika_36_F | CGCAAATGTGGCAGCTCCTTTA | 22 | Zika_36_R | CCCTTCTTCACCCAAGTAGCGA | 22 |
| Zika_37_F | AAAACACAGTCAACATGGTGCG | 22 | Zika_37_R | CCTCTTCTGGAGATCCACAGCT | 22 |
| Zika_38_F | ATGTTGTCAGGCCTGCTAGTCA | 22 | Zika_38_R | AGACCCATGGATTTCCCCACAC | 22 |
